# Apples to apples? Neural correlates of emotion regulation differences between high- and low-risk adolescents

**DOI:** 10.1093/scan/nsz063

**Published:** 2019-09-11

**Authors:** Michael T Perino, João F Guassi Moreira, Ethan M McCormick, Eva H Telzer

**Affiliations:** 1 Department of Psychiatry, Washington University in St Louis School of Medicine, 4559 Scott Avenue, Suite 1153, St Louis, MO 63110, USA; 2 Department of Psychology, University of California, Los Angeles, 502 Portola Plaza, A191 Franz Hall, Los Angeles, CA 90095, USA; 3 Department of Psychology & Neuroscience, University of North Carolina, Chapel-Hill, 235 E Cameron Avenue, Room 213D, Chapel Hill, NC 27599, USA

**Keywords:** adolescent delinquency, emotion regulation, fMRI, neurodevelopment, social processing

## Abstract

Adolescence has been noted as a period of increased risk taking. The literature on normative neurodevelopment implicates aberrant activation of affective and regulatory regions as key to inhibitory failures. However, many of these studies have not included adolescents engaging in high rates of risky behavior, making generalizations to the most at-risk populations potentially problematic. We conducted a comparative study of nondelinquent community (*n* = 24, mean age = 15.8 years, 12 female) and delinquent adolescents (*n* = 24, mean age = 16.2 years, 12 female) who completed a cognitive control task during functional magnetic resonance imaging, where behavioral inhibition was assessed in the presence of appetitive and aversive socioaffective cues. Community adolescents showed poorer behavioral regulation to appetitive relative to aversive cues, whereas the delinquent sample showed the opposite pattern. Recruitment of the inferior frontal gyrus, medial prefrontal cortex, and tempoparietal junction differentiated community and high-risk adolescents, as delinquent adolescents showed significantly greater recruitment when inhibiting their responses in the presence of aversive cues, while the community sample showed greater recruitment when inhibiting their responses in the presence of appetitive cues. Accounting for behavioral history may be key in understanding when adolescents will have regulatory difficulties, highlighting a need for comparative research into normative and nonnormative risk-taking trajectories.

## Introduction

Adolescence is often described as a paradoxical time, where relative improvements in certain domains (e.g. abstract reasoning) are often coupled with suboptimal decision making in other domains (e.g. health risk behaviors; Brener *et al.*, 2013). Much of this paradox has been attributed to the outsized role that affective processing plays in adolescents’ lives (Somerville, Hare & Casey, 2011). Specifically, adolescents’ ability to regulate their behavior is particularly affected by socioaffective cues (Chein *et al.,* 2011; Casey, 2015), which may explain the onset of negative outcomes like psychopathology (Kranzler *et al.,* 2016) and increased risk taking (Casey *et al.*, 2008; Guyer *et al.,* 2011; Nelson *et al.,* 2005; Pfeifer *et al.*, 2011; Perino *et al.*, 2016; Somerville *et al.,* 2011), and rises in delinquency (Moffitt & Caspi, 2001). Developmental neuroscience research has speculated that suboptimal behaviors like delinquency may be driven, in part, by neural changes that make adolescents more susceptible to disinhibited responses. While such work has aided in theorizing how neuroscience can inform the treatment of adolescents engaging in high rates of disruptive behaviors (Cohen & Casey, 2014; Casey *et al.,* 2017), it is unclear if these normative increases in risk taking are explanatory for the youth actively engaging in high-risk behaviors.

Neuroscientific inquiries have found socioaffective stimuli—both negatively and positively valenced—to impact regulation in adolescents, although the specific effect has not been consistent. A number of studies have found that aversive stimuli—such as fearful faces (Grose-Fifer, et al., 2014; Monk *et al.,* 2003), negative affective images (Cohen-Gilbert & Thomas, 2013), and threat cues (Dreyfuss *et al.,* 2014)—are particularly deleterious to regulatory capacities, whereas others have found that compromised regulation may be more specific to appetitive stimuli—such as happy faces (Somerville *et al.,* 2011), positive affective images (Perino *et al.,* 2016), and the presence of peers (Chein *et al.,* 2011). The nature of this discrepancy is of great importance, as it suggests adolescent dysregulation in response to socioaffective stimuli is complex and likely driven by more than merely the type of stimuli observed.

Such divergent findings may be reflective of differences in the participants’ lived experiences. For example, increased focus on appetitive socioaffective cues may theoretically help adolescents identify and act on opportunities to increase their status within their respective social hierarchies (Nelson, Jarcho, & Guyer, 2016). Not all social groups are alike, and for some adolescents, dysregulation in aversive or threatening situations may place them at an increased risk of negative consequences. Furthermore, research has shown that environmental inputs often change behavior (Bronfenbrenner, 1979), with a particular focus on the impact of unstable environments and their role in behavioral reactivity (Ellis *et al.,* 2012). What is valuable within a given environment will vary, and the socioaffective cues relevant to those consistently engaging in delinquency may fundamentally differ from their nondelinquent community counterparts. Perhaps those engaging in delinquency demonstrate behavioral and neural adaptations necessary for surviving and rising in disruptive environments (Ellis *et al.,* 2012). Alternatively, past delinquent behavior may alter processing that impacts future decision making in ways not applicable to normative samples (Agnew, 1992). To better understand how affective processing difficulties may lead some adolescents astray, more inquiries need to focus on how, when, and for whom affective stimuli are problematic, as increased generalizability to diverse samples may highlight developmental issues currently overlooked (Telzer, 2018).

The interplay between subcortical affective circuitry and regulatory cortical regions has been highlighted in accounts of adolescent disinhibition. Volumetric and functional changes in social cognition regions [e.g. the fusiform face area, superior temporal sulcus, and temporoparietal junction (TPJ); Blakemore & Mills, 2014) and increases in neural activation of affective circuitry (e.g. amygdala (Guyer *et al.,* 2008), ventral striatum (Galvan, 2010)], coupled with aberrant activity in regulatory regions [e.g. inferior frontal gyrus (IFG), medial prefrontal cortex (mPFC); Crone & Dahl, 2012], are reported in functional magnetic resonance imaging (fMRI) research with adolescent samples. These neural changes are proposed to reorient cognitive resources toward salient socioaffective cues and away from regulatory processing (Nelson *et al.,* 2005; Somerville *et al.,* 2011; Nelson *et al.,* 2016; Perino *et al.,* 2016). The amygdala and ventral striatum show greater responsivity to appetitive socioaffective cues in adolescence, which may override inhibition (Somerville *et al.,* 2011; Perino *et al.,* 2016). The recruitment of prefrontal regions implicated in executive functioning [such as the IFG (Aron, Robbins & Poldrack., 2014) and the mPFC (Dreyfuss *et al.,* 2014)] is necessary to successfully inhibit and override the attentional resources engaged in the presence of salient affective cues (Theeuwes, 2010). Given the role these regulatory regions have shown in successful inhibition in the presence of salient stimuli (Serences *et al.,* 2005; Sharp *et al.,* 2010; Swann *et al.,* 2012; Aron *et al.,* 2014), they likely play a key role in adolescents’ ability to successfully focus on the task at hand and away from distracting salient information (Fukada & Vogel, 2009; McCormick & Telzer, 2017; McCormick *et al.*, 2016).

In the current study, a low-risk community and high-risk delinquent sample of adolescents completed a modified go/no-go task (Cohen-Gilbert & Thomas, 2013) during which participants were instructed to inhibit a prepotent behavioral response while distracted by socioaffective cues, which were either appetitive or aversive social stimuli. We assessed how the presence of these cues differentially impacted inhibitory and neural responses. By comparatively assessing the influence of socioaffective cues on both delinquent and community adolescents, we aimed to extend normative neurodevelopmental work into a high-risk population. There is a need to test whether regulatory problems observed in delinquent adolescents are different from their community peers in terms of the magnitude (i.e. same patterns of behavior and brain across appetitive and aversive contexts, but higher overall deficits in the delinquent sample) or context (i.e. different patterns of behavior and brain across appetitive and aversive contexts) in order to correctly conceptualize disinhibition leading to psychosocial dysfunction.

On the one hand, current theory suggests social reorientation may impact all adolescents along a continuum (Nelson *et al.,* 2016), such that the patterns of behavioral and neural processes are pointed in the same direction, in which case delinquent adolescents may represent more extreme examples of disinhibition relative to their nondelinquent community counterparts (Young *et al.,* 2009). However, we argue that, rather than showing overall greater difficulty to the same cues, delinquent adolescents’ disinhibition may differ based on the socioaffective context they are experiencing. Adolescents with behavioral issues may have adapted to their environment in such a fashion that social reorientation directs attention to cues of threat or those that evoke aversive affective states. Thus, adolescents engaging in disruptive behaviors may find aversive cues more salient, as this may signify oncoming danger, whereas appetitive cues may be more salient for nondelinquent samples, as processing these cues may provide adolescents opportunities to rise in their social hierarchy. In other words, the process of a social reorientation may be universal, in that attentional resources at this developmental stage are disproportionately directed toward socioaffective cues in the environment (Nelson *et al.,* 2016), but the cues that are salient may differ based on life experience and behavioral profile. Clarifying the nature of inhibitory responses to socioaffective cues in adolescence may provide valuable insight into explaining what differentiates those undergoing normative development from those actively engaged in high-risk behaviors.

In the current study, we hypothesized that socioaffective cues would lead to greater recruitment of attentional resources (Theeuwes, 2010) and greater disruption in task performance in an implicit emotion regulation task. We predicted that the emotion regulation difficulties observed in the task would depend on the behavioral profiles (low-risk community, high-risk delinquent) of the adolescents. Specifically, community youth will show greater disruption to appetitive cues (Perino *et al.,* 2016), while high-risk youth will show greater disruption to aversive cues (Casey *et al.,* 2017).

## Methods

### Participants

A total of 51 adolescents were recruited, with a total of 48 adolescents included in the final sample after removing participants unsuitable for analyses [one participant was removed due to an inability to adequately complete the scanning protocol (as determined by poor task comprehension during training and excessive movement throughout the scanning protocol), and two others chose to not complete the scanning session]. The community and delinquent sample each included 24 participants (see Table 1 for demographic breakdown of both samples). Given that disciplinary contacts with school (e.g. suspensions and expulsions) and legal institutions (e.g. arrests) in adolescence have been linked with continued criminal behavior (Wald & Losen, 2003; Weiner, 2003; Rocque & Paternoster, 2011), we recruited participants engaging in antisocial behaviors that resulted in institutional involvement (e.g. property theft, fighting, drug use and/or sale, weapon use, etc. leading to disciplinary actions). We recruited the delinquent sample from (i) an alternative school for students who have been expelled or suspended (ii), the local juvenile detention center, and (iii) the local parole and probation office. To provide greater clarity regarding the scope of delinquency and institutional discipline, the numbers of suspensions, expulsions, and arrests were collected in the delinquent sample (Table 2). Participants in the community sample were recruited from traditional schools in the same geographic area. Participants were compensated US $50. Informed consent and assent were obtained for participants in accordance with the university’s institutional review board.

**Table 1:** Demographic information for community and delinquent adolescents

	Community sample (*n* = 24)	Delinquent sample (*n* = 24)
Age, mean (SD), range	15.8 (.36), 15.5–16.5	16.2 (1.2), 13.1–17.8
No. female	12	12
No. white	17	12
No. black	3	12
No. other ethnicity	4	0

**Table 2:** Disciplinary history of delinquent sample

	No. of times disciplinary act occurred							
	1	2	3	4	5	6	7	8+
Suspensions	8.3%	16.7%	25%	0%	4.2%	0%	0%	41.7%
Expulsions	41.7%	12.5%	4.2%	0%	0%	0%	0%	0%
Arrests	25%	8.3%	0%	4.2%	4.2%	0%	8.3%	0%

### Experimental paradigm: Go/No-Go and social Go/No-Go task

While completing an fMRI scan, participants completed both a control go/no-go, which was used solely to establish baseline cognitive performance in the absence of socioaffective cues, and a social go/no-go task (Perino *et al.,* 2016) adapted from prior research (Cohen-Gilbert & Thomas, 2013), which was used to assess emotion regulation in the context of socioaffective cues. The control go/no-go task consisted of four blocks, each containing 25 trials. The control task was completed prior to the social go/no-go, which included four aversive and four appetitive blocks, which were presented in a randomized order. Participants were presented with blocks of socially appetitive or aversive scenes for 300 ms, after which a letter was superimposed on the image for 500 ms. During this 500 ms window, participants were instructed to respond as quickly as possible by pushing a button for every letter shown (‘go’) except the letter ‘X’ (‘no-go’). The control go/no-go task was identical in design structure, but did not include superimposed images (rather, a white square was presented on a black screen for 300 ms, after which a black letter was superimposed on the white background for 500 ms). In both task variants, 28% of the trials were no-go trials, which created a prepotent response to press, requiring inhibition on no-go trials. A jittered Intertrial interval (ITI) was presented between trials, averaging 1200 ms. In total, the social go/no-go consisted of 100 trials per condition across eight randomized blocks. Socially appetitive blocks included scenes of people celebrating, cooperating, and being affiliative, while socially aversive blocks included scenes of people excluding one another, bullying peers, and showing negative affect (see Perino *et al.,* 2016 for selection and reliability of the stimuli). The task was programmed and presented using E-Prime 2.0 (2012).

To assess for behavioral performance, we chose to use *d*’ as our behavioral measure of emotion regulation (Cohen *et al.,* 2016). *d*’ is an index originating from signal detection theory in which the normalized rate of correct discrimination of a signal (‘hits’) is compared to the normalized rate of false attributions of signal due to noise (‘false alarms’) (Green & Swets, 1966; Stanislaw & Todorov, 1999). Given that socioaffective stimuli may alter a number of psychological processes, *d*’ is an ideal metric given its incorporation of multiple signal (correct hits and correct inhibition) and noise (incorrect misses and false alarms) components. In this experiment, a ‘hit’ was defined as making a button press when appropriately required to (i.e. on go trials), and a false alarm was defined as making a button press when inappropriate to do so (i.e. on no-go trials). *d*’ was calculated for each participant by subtracting their normalized (*Z*) score of false alarm rates from their normalized (*Z*) score of hit rates; thus, *d*’ for each condition equaled *Z*_Hit_ − *Z*_False Alarm_ (Macmillan & Creelman, 1991). *d*’ was calculated separately for socially appetitive and socially aversive blocks as well as the baseline control task. Calculating *d*’ within condition provided an index of how well participants performed with and without affective stimuli, where greater *d*’ values indicate better performance on the task (i.e. more effective inhibition coupled with less disinhibition). Utilizing a control go/no-go is optimal for establishing baseline rates of inhibitory control. Given that emotional stimuli are more engaging than the control condition, and our stated focus on how adolescent decision making is specifically affected in social contexts (Nelson *et al.,* 2016), we examined how socioaffective cues (appetitive versus aversive) may have distinct effects on emotion regulation on the social go/no-go.

### fMRI data acquisition

Imaging data were collected with a 3 T Siemens TRI MRI scanner. T2^*^-weighted, matched-bandwidth, high-resolution anatomical scan [repetition time (TR) = 4 s; echo time (TE) = 64 ms; field of view (FOV) = 230; matrix = 192x192; slice thickness = 3 mm; 38 slices], and T1^*^ magnetization-prepared rapid-acquisition gradient echo (MPRAGE; TR = 1.9 s; TE = 2.3 ms; FOV = 230; matrix = 256 × 256; sagittal plane; slice thickness = 1 mm; 192 slices) scans were acquired as structural images. Each condition of the experimental paradigm comprised 120 T2-weighted echoplanar images (EPI; slick thickness = 3 mm, 38 slices; TR = 2 s; matrix = 92x92; FOV = 230 mm; voxel size of 3x3x3 mm). An oblique axial orientation was used to maximized coverage area and reduce signal dropout for the T2 images.

**Fig. 1:**
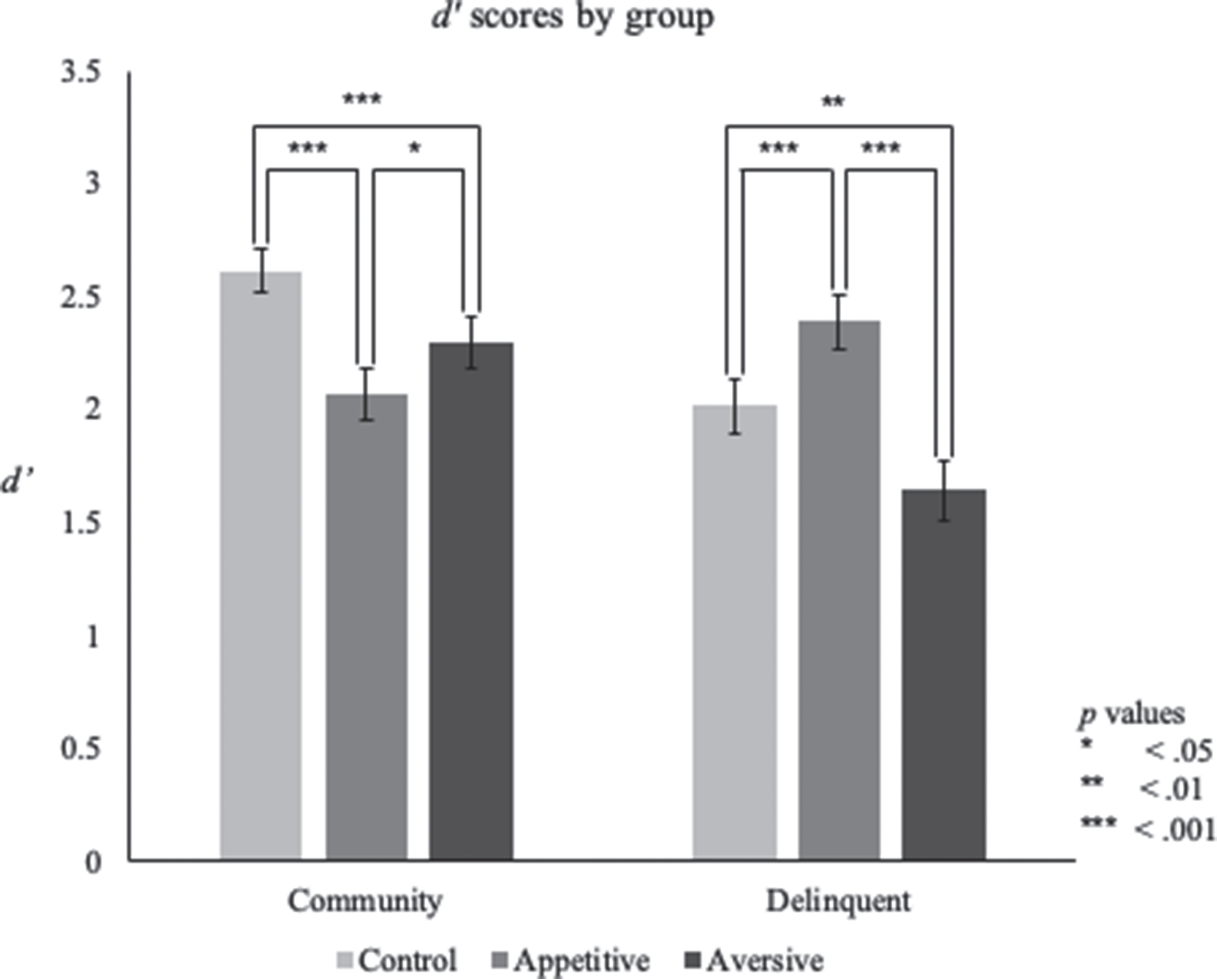
Within-group comparisons of *d*’ as a function of task condition

### fMRI data preprocessing and analysis

Data preprocessing and analysis were conducted using Statistical Parametric Mapping (SPM8; Wellcome Trust Centre for Neuroimaging, University College, London, UK). Functional images were spatially realigned in order to correct for movement (no participant exceeded 3 mm of maximum image to image motion in any direction for more than 5% of their echoplanar images). The images were coregistered to each participant’s high-resolution MPRAGE and segmented into cerebrospinal fluid, gray matter, and white matter. A normalization transformation matrix was applied to the functional and T2 structural images, thereby converting each participant’s data into the standard stereotactic space specified by the Montreal Neurological Institute. Normalized functional data were smoothed using an 8-mm Gaussian kernel (full width at half maximum). A restricted maximum likelihood algorithm, with an autoregressive model order of 1, was used to address serial autocorrelations, and a high-pass filter with a 128-s cutoff was applied to remove low-frequency noise.

Data were analyzed using the general linear model (GLM) in the SPM software. At the individual level, a fixed-effects analysis was modeled with a block design, in which all individual trials were modeled within each block (800-ms duration) for each condition (socially appetitive, socially aversive), so that null events (i.e. jittered ITIs) served as the implicit baseline. To model inhibitory processing, a parametric modulator (PM) was included for each trial in participants’ first level model for the conditions of interest (appetitive or aversive socioaffective cues) to represent behavioral accuracy. We represented successful completion of an individual trial, such that 1 = correct response (correct hit or ‘go’ and correct inhibition or ‘no-go’) and 0 = incorrect response (incorrect hit or ‘false alarm’ and not responding on go trials ‘miss’). The PM isolates neural responses linked to behavioral performance on the task (i.e. successful inhibition relative to failed inhibition), allowing us to identify regions specifically recruited for successful relative to unsuccessful behavioral performance. Significant voxels represent brain regions that show parametrically greater activation to correct versus incorrect trials based on the given condition of interest (socially appetitive or socially aversive).

Parameter estimates from the GLM were used to create linear contrasts for comparisons of interest (socially appetitive > socially aversive) at the group level. Random-effects, whole-brain analyses were conducted in order to examine group differences between delinquent and community adolescents. To correct for multiple comparisons at the group level, we conducted a Monte Carlo simulation using the AFNI software package’s 3dClustSim command for the group-level brain mask (Ward, 2000) and corrected for intrinsic smoothing, which was estimated using the 3dFWHMx command and acf. Results of the simulation indicated that a family-wise error–corrected rate of *P* < 0.05 would be achieved with a voxel-wise threshold of *P* < 0.005 and a minimum cluster size of 132 voxels. Age, gender, and ethnicity (white, black, and other) were controlled in all analyses. All neural analyses are available on Neurovault (https://neurovault.org/collections/4081/).

## Results

### Behavioral results

To examine behavioral differences between the low-risk community and high-risk delinquent samples, we used SPSS (2017) to conduct a repeated-measures GLM with one within-subject variable (task condition: control, appetitive, aversive) and one between-subject variable (group: community, delinquent), while controlling for self-reported ethnicity, age, and gender. The effects of task (*F*(2,74) = .128) and group (*F*(1,37) = 2.548) were not statistically significant, but there was a significant condition-by-group interaction (*F*(1,74) = 13.419, *P* < 0.001, η_p_^2^ = .266). We used paired-samples *t*-tests to explore the group-by-task interaction effect (Figure 1). In the community sample, inhibitory performance in the control condition (*d*’_Mean_ = 2.617, SD = 0.475) was significantly better than in both the appetitive (*d*’_Mean_ = 2.07, SD = 0.537, (*t*(23) = 4.18, *P* < 0.001, Cohen *d* = 0.853) and aversive condition {*d*’_Mean_ = 2.299, SD = 0.541, [*t*(23) = 2.884, *P* = 0.008, Cohen *d* = 0.589]}; additionally, inhibitory performance in the aversive condition was significantly better than in the appetitive condition [*t*(23) = 2.079, *P* = 0.049, Cohen *d* = 0.424]. Inhibitory performance patterns in the delinquent sample differed from the community sample. Specifically, inhibitory performance in the appetitive condition (*d*’_Mean_ = 2.391, SD = 0.591) was significantly better than in both the control (*d*’_Mean_ = 2.017, SD = 0.567, (*t*(23) = 3.747, *P* = 0.001, Cohen *d* = 0.765) and aversive condition (*d*’_Mean_ = 1.647, SD = 0.644, (*t*(23) = 9.469, *P* < 0.001, Cohen’s *d* = 1.932); additionally, inhibitory performance in the control condition was significantly better than in the aversive condition (*t*(23) = 3.318, *P* = 0.003, Cohen *d* = 0.678). In short, the community sample showed greater inhibitory failures to appetitive cues, whereas the delinquent sample showed greater inhibitory failures to aversive cues. These findings suggest emotion-regulation difficulties for members of each group differ based on the socioaffective context, as the delinquent sample shows a different pattern of emotion regulation disruption compared to their community counterparts.

**Table 3:** Neural regions that differentiate group (delinquent, community) and socioaffective condition (socially appetitive > socially aversive) linked to successful task performance

Region	*t*	*k*	*x*	*y*	*z*
mPFC	4.31	349	6	41	−11
R IFG	4.06	168	30	26	−11
L IFG	4.69	336	−42	35	−2
L TPJ	3.66	176	−45	−76	37
L cuneus	3.82	269	−12	−67	28
Brainstem	4.89	195	−6	−13	−17

Note. R refers to right and L refers to left; *x*, *y*, and *z*, to Montreal Neurological Institute coordinates; *t*, *t*-score at those coordinates (local maxima); *k*, number of contiguous voxels; IFG, inferior frontal gyrus; mPFC, medial prefrontal cortex; TPJ, temporal parietal junction.

### Neuroimaging results

**Fig. 2:**
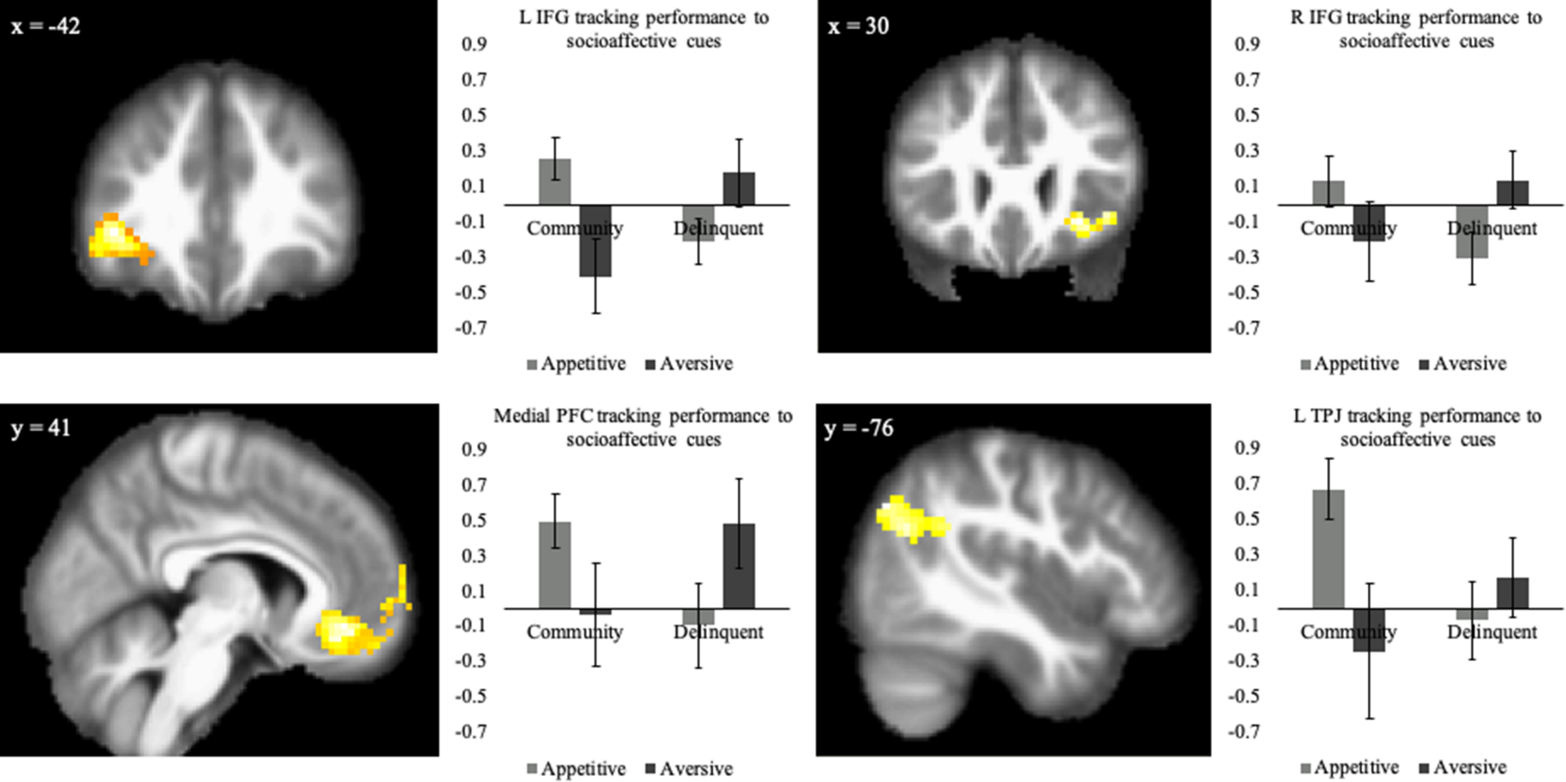
Community and delinquent adolescents show differential tracking of successful inhibition in the left (L) and right (R) inferior frontal gyrus (IFG), medial prefrontal cortex (PFC), and left temporal parietal junction (TPJ). The *y* axis represents parameter estimates of signal intensity from neural regions that tracked with successful task performance. Positive numbers indicate increased recruitment in each region when successfully performing, whereas numbers around 0 indicate the region was not recruited as a function of task performance.

Given that both behavioral profile and cue type produced a significant interaction effect on behavioral disruption, we next examined regions tied to successful task completion between groups. We conducted a two-sample (delinquent vs. community) whole-brain *t*-test comparing neural activation on the main contrast of interest (socially appetitive cues > socially aversive cues), using the PM for successful task completion relative to task failures. We observed significant group differences in the bilateral IFG, mPFC, TPJ, cuneus, and brainstem (Table 3). To understand the nature of interaction, we extracted parameter estimates from the IFG, mPFC, and TPJ clusters to unpack how these regions differentiated inhibitory success as a function of group and cue type. For descriptive purposes, we extracted parameter estimates of signal intensity from each group separately within the aversive and appetitive blocks. As shown in Figure 2, delinquent adolescents showed greater recruitment during successful inhibition toward socially aversive cues compared to community adolescents who showed greater recruitment during successful inhibition toward socially appetitive cues, suggesting that recruitment of these regions promoted successful task performance during the cue type where each group showed the most inhibitory failures. This suggests that the IFG, mPFC, and TPJ are involved in successfully inhibiting behavior in the presence of socioaffective cues; however, recruitment is not uniformly observed for all socioaffective cues but depends on the types of cues that underlie emotion-regulation difficulties for each group. Looking solely within the control condition, we see significant activation differences in the posterior cingulate cortex; for the purposes of this article, focused on social cognition, we do not explore this further, although the analysis can be found on Neurovault (https://neurovault.org/images/129030/). Additionally, we conducted an exploratory seed-based functional connectivity analysis using the clusters of activation observed in the mPFC and the IFG. We conducted whole-brain connectivity analyses comparing the two groups using the gPPI toolbox in SPM (McLaren *et al.,* 2012). We did not observe significant differences between the two groups in connectivity with either region that survived multiple-comparisons threshold correction.

## Discussion

Adolescence is a transitory period, where social information takes on great importance for achieving age-specific goals (Nelson *et al.,* 2016). The increased focus on socioaffective information guides attention toward cues that may signify a window of opportunity for adolescents to rise socially (Crone & Dahl, 2012). However, these opportunities for social advancement may also bear increased risk, and when neural reactivity is coupled with poor regulation, suboptimal outcomes may result (Casey, 2015). Previous research examining the effects of socioemotional stimuli on adolescents has shown that appetitive cues (Somerville *et al.,* 2011; Perino *et al.,* 2016) are linked with disinhibition at the behavioral and neural level in normative adolescent development. To extend this research to at-risk populations, we compared a community sample to a delinquent sample of adolescents to assess if these groups responded to socioaffective cues similarly (with the main distinction being one of magnitude where delinquent adolescents may show more difficulty with emotion regulation) or if these groups may respond differentially (with delinquent youth showing different patterns of emotion regulation difficulties). We found that the emotion regulation differences between community and delinquent adolescents were not entirely one of magnitude, as community adolescents showed greater difficulties in the presence of appetitive social cues, whereas delinquent adolescents showed greater difficulties in the presence of aversive social cues.

At the neural level, we observed that recruitment of the IFG, mPFC, and TPJ distinguished the groups. Delinquent adolescents showed significantly greater recruitment of these regions when successfully inhibiting their responses specifically in the presence of aversive cues, whereas the community sample showed greater recruitment in these regions when successfully inhibiting their responses in the presence of appetitive cues. These results suggest that while the same regulatory and social processing regions were recruited during the task, when they were recruited, they differed depending on the group (delinquent, community) and condition (aversive, appetitive). The inclusion of these regions is noteworthy, as they have been implicated in much of the developmental social neuroscience research examining adolescent cognition in social contexts (Hoorn *et al.,* 2019).

Interestingly, recruitment of the IFG and mPFC is greatest when it was most difficult for adolescents to complete the task without error. In particular, community adolescents showed the greatest behavioral disruption to appetitive socioaffective cues, which coincided with greater IFG recruitment, whereas delinquent adolescents showed the greatest behavioral disruption to aversive socioaffective cues, which coincided with greater IFG recruitment. The IFG has been consistently identified as a region implicated in inhibiting behavioral responses broadly (Swick *et al.*, 2008; Hampshire *et al.,* 2010), as well as tracking salient information and guiding attentional resources (Aron *et al.,* 2004; Menon & Uddin, 2010; Aron *et al.,* 2014). Hence, it is not surprising that the ability to focus on the go/no-go task while in the presence of affective stimuli was linked with IFG recruitment, as this would rely on inhibiting a disruptive affective response, focusing on task-specific instructions, and guiding attentional resources away from distracting cues.

The mPFC has been implicated in suboptimal adolescent decision making, specifically in relation to impulsivity (Dreyfuss *et al.,* 2014) and disruption related to affective stimuli (Dixon *et al.,* 2017). The mPFC is also linked with tracking errors during cognitive control (McCormick & Telzer, 2017), as well as negative feedback during risk taking (McCormick & Telzer, 2018), suggesting that the mPFC may be integral in decision making. Together, our findings suggest that the mPFC aids in monitoring errors and task-irrelevant information, as greater recruitment of this region corresponds to improved performance in conditions that the adolescents found the task most challenging. Ostensibly, the IFG and mPFC were recruited to regulate distracting cues and complete the task; however, when recruitment occurred, they differed between the adolescent groups depending on the socioaffective cues.

A similar pattern was found for the TPJ. The TPJ is implicated in many processes, including social cognition (Saxe, 2006). In adolescence, TPJ activation is tied to observing social cues (Burnett *et al.,* 2009) and is theorized to be integral in increases in social information processing observed in this time period (Nelson *et al.,* 2005). While some studies have linked increased TPJ activation with decreased risk taking (Guassi Moreira & Telzer, 2018) and increased prosocial decision making (van Hoorn *et al.,* 2016), others have implicated decreased recruitment of the TPJ in improved task performance (McCormick & Telzer, 2017), and it is consistently activated in adolescent decision making in social contexts (Hoorn *et al.,* 2019). Perhaps the recruitment of the TPJ in the current study is indicative of greater social information processing or attentional capture (Vossel *et al.*, 2014). Given that community adolescents needed greater regulatory recruitment during the appetitive condition—and vice versa for the delinquent adolescents—it would stand to reason that this recruitment was in response to the adolescents more fully processing cues in the given condition. As our results show, clarifying the role the TPJ plays in social information processing and how that impacts regulatory mechanisms is important in addressing (sub)optimal behaviors in adolescence.

The differential recruitment of regulatory and social information processing regions in this task suggests that delinquent and community adolescents may fundamentally differ in their processing of socioaffective cues. The presence of affective stimuli is irrelevant to the task behavior (i.e. each subject is supposed to either press or inhibit their response to the letter stimuli, regardless of the background image); however, our results highlight that task-irrelevant affective information can have an outsized role for adolescents. While models of adolescent brain development have postulated that individual difference factors (such as life history and environmental context) likely alter how socioaffective and regulatory systems evolve (Nelson *et al.,* 2016), such positions until recently have been mostly theoretical (Foulkes & Blakemore, 2018; Lee *et al.,* 2018; Perino *et al.*, 2019).

Our research highlights that while emotional stimuli may be problematic across adolescence, the effect is not uniform and is distinguishable when accounting for behavioral characteristics. Community adolescents had problems with appetitive stimuli—perhaps reflective of disruption in approach to the presence of prosocial cues—while delinquent adolescents had problems with aversive stimuli—perhaps reflective of disruption toward threat or environmental instability, which may be more prevalent in their daily lives (Agnew, 1992; Ellis *et al.,* 2012). Our task utilized emotional stimuli to indirectly capture attention, requiring adolescents to engage regulatory processing to redirect the adolescent (e.g. the distracting effect of emotion). Tasks that require more explicit focus on emotional stimuli for task completion, such as reading emotional cues, likely invoke different psychological processes (Lee *et al.,* 2019; McCormick *et al.*, 2018), a distinction that may be particularly useful for pinpointing when delinquent adolescents differ from their low-risk counterparts. Historically, studies examining adolescents with conduct issues have observed a broad pattern of physiological hypoactivity (as measured by heart rate, galvanic skin response, eye-blink startle, and neuroimaging studies) to negatively valenced stimuli (Blair *et al.*, 2016). The findings presented in this study suggest future research should examine how socioaffective information may be incorporated into decision-making processes irrespective of physiological responsivity, as emotional stimuli are likely to impact behavior even if it does not impact autonomic functioning. Our results provide preliminary evidence to the hypothesis that behavioral profiles may undergird neurodevelopment, as the past behavior of adolescents was related to both when they were likely to be distracted by affective stimuli, and when the same neural regions were recruited to successfully regulate behavior.

We found that past delinquent acts were a meaningful variable for understanding emotion regulation, as we saw differential neurobehavioral responses. High-risk adolescents’ problematic behaviors may be tied to adapting to aversive social cues and contexts, which suggests that assessing the contextual information surrounding past behavior may be integral to understanding delinquency and sharpening future research inquiries. Disinhibition, in and of itself, is not a universally problematic phenomenon, as acting quickly in response to seeing opportunities for rewarding social gains or threatening contexts may sometimes be beneficial. As the field of developmental neuroscience progresses, the need to apply our findings within the greater context of varying social structures becomes more pertinent (Casey *et al.,* 2016). In order for empirically based findings to best inform potential interventions aimed at addressing social problems, it is imperative that we base our results on samples actively engaged in the problems we want to tackle (e.g. delinquency) in relevant situational contexts. Our study suggests that ancillary socioaffective cues are likely to be particularly distracting in adolescence. Future work ought to tackle how disinhibition to varying social stimuli may directly (or indirectly) connect to substance use and antisociality, as the relationship between social disinhibition and antisocial behaviors occurring due to psychopathology is currently not well elucidated. Exploring if these suboptimal outcomes are driven by, or orthogonal to, socially provoked disinhibition will be of great importance for future research.

While this study represents an important step toward understanding how individual difference factors need to be accounted for in developmental neuroscience, there are limitations that require future attention. First, because we used a cross-sectional design, we did not explore the progression of emotion regulation in adolescents, which limits our ability to developmentally answer how regulatory ability changes due to learned experience (Agnew, 1992; Ellis *et al.,* 2012). It is possible that such experiences may alter how individuals perceive socioaffective stimuli in future interactions, a concern we are unable to address in the current study. Future research should examine how individual difference factors (such as behavioral history) alter trajectories of affective perception and the progression of emotion regulation difficulties. By applying both prospective and retrospective analyses that tie neural development to meaningful behavioral differences, researchers may gain insight into critical intervention periods. Future research examining how the development of social cognition is impacted by behavior and the environmental inputs, as well as how that might cascade into the neurodevelopment of regulatory processing, is of the most importance.

Second, while the distinction between adolescents who offend and those who do not has been shown to be quite meaningful (Cohen & Piquero, 2009; Cohen *et al.*, 2010; Moffitt & Caspi, 2001), this assessment is somewhat blunt and oftentimes open to class, gender, and race biases (Wald & Losen, 2003). We attempted to mitigate this concern by selecting a gender- and race-balanced cohort for our high-risk delinquent sample that was from the same region as our community sample, but we recognize that not all acts receive the same type of discipline and that not all disciplinary actions are inherently just or represent equivalently severe transgressions. We believe verifiable evidence of a disciplinary act would be a useful starting point but recognize the need for greater specificity of transgression in future endeavors. While having a continuous measure of verifiable acts of delinquency would be ideal for such a study, verifying such information without secondary sources (e.g. school, police, and social services records), clinical assessments, and parental input is quite difficult. There have been calls for greater specificity and standardization when assessing delinquency, such as focusing on intent, motivational factors, and specific behaviors present in delinquent acts (Welner *et al.,* 2018); perhaps this is an avenue the field can proceed down to improve the precision of our findings to the detrimental behaviors delinquent populations may engage in. Quantifying delinquent acts on a number of continuums will allow for greater precision in explaining physiological differences and targets for intervention in future research.

Third, we did not have clinical assessments or other measures aimed at delinquency collected in both groups; future work should incorporate both verifiable evidence and a wide assay of individual difference factors, as variations in social motivation, perceptual processing, and lived experience are theorized to explain much of when and why adolescents focus on socioaffective stimuli (Nelson *et al.,* 2016). There is a large body of research focusing on externalizing and aggression (see Blair *et al.*, 2014; Blair *et al.*, 2016; Viding & McCrory, 2012) that has by proxy examined delinquent behavior via clinical populations. While certainly valuable, this research base may not be dispositive toward understanding normative increases in adolescent risk taking and how that can result in suboptimal outcomes in nonclinical samples. Part of the complication is that much of the aforementioned research focuses on deficiencies in offending youth (Bjorklund & Hawley, 2014). Focusing on antisocial behaviors versus psychometrically assessed constructs associated with antisociality provides different information that may muddy how we understand delinquency and offending populations (Hyde *et al.*, 2013). Given that research into hierarchical approaches of psychopathology has found that delinquent outcomes are tied to both general dysfunction (the p-factor) and externalizing syndromes (Caspi *et al.,* 2014), measurement issues and outcome convergence need to be thoughtfully addressed in future research (Watts *et al.*, 2019). Future research into adolescent risk taking ought to focus more on recruiting delinquent samples, increased collection of demographic and individual difference variables, and providing as much contextual information regarding past behavior as feasible. Many studies, including this one, are limited by their sample size and time requirements to get fine-grained assessments across individual differences.

Our results help to shed light on the process of successful emotion regulation in adolescents, as well as provide a window into how prior behavior may inform when and why adolescents are likely to engage in specific types of suboptimal decision making. The cues that lead adolescents without prior histories of delinquency astray may not be equivalent for adolescents already engaging in delinquent acts. Emotion regulation difficulties across different adolescents are specific to both behavioral history and social context, and research surrounding how to best understand and ultimately intervene upon problematic adolescent outcomes ought to account for this discrepancy. This study provides a useful comparative analysis, showing that emotion regulation failures seen across adolescence require accounting for behavior profile, as community adolescents’ emotion regulation difficulties were qualitatively different from delinquent adolescents.

## Funding

This manuscript was partially supported by grants from the National Science Foundation (NSF SES 1459719 to E.H.T. and NSF Graduate Fellowship 2 016 220 797 to J.F.G.M.), the National Institute of Drug Abuse (R01DA039923 to E.H.T.), the National Institute of Mental Health (NIMH 2T32MH100019-06 to J.L.L. & D.M.B.) and generous funds from the Department of Psychology at the University of Illinois. All authors report no biomedical financial interests or potential conflicts of interest.

## References

[ref1] Agnew R. (1992). Foundation for a general strain theory of crime and delinquency. Criminology, 30(1), 47–88.

[ref2] Anderson B.A., Laurent P.A., Yantis S. (2011). Value-driven attentional capture. Proceedings of the National Academy of Sciences, 108(25), 10367–71. 10.1073/pnas.1104047108 PMC3121816 21646524

[ref3] Aron A.R., Robbins T.W., Poldrack R.A. (2004). Inhibition and the right inferior frontal cortex. Trends in Cognitive Sciences, 8(4), 170–7. 15050513 10.1016/j.tics.2004.02.010

[ref4] Aron A.R., Robbins T.W., Poldrack R.A. (2014). Inhibition and the right inferior frontal cortex: one decade on. Trends in Cognitive Sciences, 18(4), 177–85. 24440116 10.1016/j.tics.2013.12.003

[ref5] Bao W.N. (2017). Societal-Level Changes and Criminogenic Strain In: In Delinquent Youth in a Transforming China, Cham: Palgrave Macmillan, pp. 29–69.

[ref6] Bjorklund D., Hawley P.H. (2014). Aggression grows up: Looking through an evolutionary developmental lens to understand the causes and consequences of human aggress In: The Evolution of Violence, New York, NY: Springer, pp. 159–86.

[ref7] Blair R.J.R., Leibenluft E., Pine D.S. (2014). Conduct disorder and callous–unemotional traits in youth. New England Journal of Medicine, 371(23), 2207–16. 25470696 10.1056/NEJMra1315612 PMC6312699

[ref8] Blair R.J.R., Veroude K., Buitelaar J.K. (2016). Neuro-cognitive system dysfunction and symptom sets: a review of fMRI studies in youth with conduct problems. Neuroscience & Biobehavioral Reviews, 91, 69–90 27794436 10.1016/j.neubiorev.2016.10.022

[ref9] Blakemore S.J., Mills K.L. (2014). Is adolescence a sensitive period for sociocultural processing? Annual Review of Psychology, 65, 187–207. 10.1146/annurev-psych-010213-115202 24016274

[ref10] Brener N.D., Kann L., Shanklin S., Kinchen S., Eaton D.K., Hawkins J., Flint K.H. (2013). Methodology of the youth risk behavior surveillance system−2013. Morbidity and Mortality Weekly Report: Recommendations and Reports, 62(1), 1–20. 23446553

[ref11] Bronfenbrenner U. (1979). The ecology of human development: experiments by nature and design. American Psychologist, 32, 513–31.

[ref12] Burnett S., Bird G., Moll J., Frith C., Blakemore S.J. (2009). Development during adolescence of the neural processing of social emotion. Journal of Cognitive Neuroscience, 21(9), 1736–50. 18823226 10.1162/jocn.2009.21121 PMC4541723

[ref13] Cairns R.B., Cairns B.D., Neckerman H.J., Gest S.D., Gariepy J.L. (1988). Social networks and aggressive behavior: peer support or peer rejection? Developmental Psychology, 24(6), 815–23.

[ref14] Casey B.J. (2015). Beyond simple models of self-control to circuit-based accounts of adolescent behavior. Annual Review of Psychology, 66, 295–319. 10.1146/annurev-psych-010814-015156 25089362

[ref15] Casey B.J., Jones R.M., Hare T.A. (2008). The adolescent brain. Annals of the New York Academy of Sciences, 1124(1), 111–26. 18400927 10.1196/annals.1440.010 PMC2475802

[ref16] Casey B.J., Galván A., Somerville L.H. (2016). Beyond simple models of adolescence to an integrated circuit-based account: a commentary. Developmental Cognitive Neuroscience, 17, 128–30. 26739434 10.1016/j.dcn.2015.12.006 PMC6987976

[ref17] Casey B. J., Bonnie R. J., Davis A., et al. (2017). How should justice policy treat young offenders? A knowledge brief of the MacArthur Foundation Research Network on Law and Neuroscience.

[ref18] Caspi A., Houts R.M., Belsky D.W., et al. (2014). The p factor: one general psychopathology factor in the structure of psychiatric disorders? Clinical Psychological Science, 2(2), 119–37. 25360393 10.1177/2167702613497473 PMC4209412

[ref19] Chein J., Albert D., O’Brien L., Uckert K., Steinberg L. (2011). Peers increase adolescent risk taking by enhancing activity in the brain’s reward circuitry. Developmental Science, 14(2), 1–10. 21499511 10.1111/j.1467-7687.2010.01035.x PMC3075496

[ref20] Cohen A.O., Casey B.J. (2014). Rewiring juvenile justice: the intersection of developmental neuroscience and legal policy. Trends in Cognitive Sciences, 18(2), 63–5. 24480533 10.1016/j.tics.2013.11.002

[ref21] Cohen M.A., Piquero A.R. (2009). New evidence on the monetary value of saving a high-risk youth. Journal of Quantitative Criminology, 25(1), 25–49.

[ref22] Cohen M.A., Piquero A.R., Jennings W.G. (2010). Studying the costs of crime across offender trajectories. Criminology & Public Policy, 9(2), 279–305.

[ref23] Cohen A.O., Breiner K., Steinberg L., et al. (2016). When is an adolescent an adult? Assessing cognitive control in emotional and nonemotional contexts. Psychological Science, 27(4), 549–62. 26911914 10.1177/0956797615627625

[ref24] Cohen-Gilbert J.E., Thomas K.M. (2013). Inhibitory control during emotional distraction across adolescence and early adulthood. Child Development, 84(6), 1954–66. 23506340 10.1111/cdev.12085 PMC3688699

[ref25] Crone E.A., Dahl R.E. (2012). Understanding adolescence as a period of social–affective engagement and goal flexibility. Nature Reviews Neuroscience, 13(9), 636–50. 22903221 10.1038/nrn3313

[ref26] DeLisi M., Gatling J. (2003). Who pays for a life of crime? An empirical assessment of the assorted victimization costs posed by career criminals. Criminal Justice Studies, 16(4), 283–93.

[ref27] Dixon M.L., Thiruchselvam R., Todd R., Christoff K. (2017). Emotion and the prefrontal cortex: an integrative review. Psychological Bulletin, 143(10), 1033–81. 28616997 10.1037/bul0000096

[ref28] Dreyfuss M., Caudle K., Drysdale A.T., et al. (2014). Teens impulsively react rather than retreat from threat. Developmental Neuroscience, 36(3–4), 220–7. 24821576 10.1159/000357755 PMC4125471

[ref29] Ellis B.J., Del Giudice M., Dishion T.J., et al. (2012). The evolutionary basis of risky adolescent behavior: implications for science, policy, and practice. Developmental Psychology, 48(3), 598–623. 22122473 10.1037/a0026220

[ref30] Foulkes L., Blakemore S.J. (2018). Studying individual differences in human adolescent brain development. Nature Neuroscience, 21(3), 315–323. 29403031 10.1038/s41593-018-0078-4

[ref31] Fowler C.H., Miernicki M.E., Rudolph K.D., Telzer E.H. (2017). Disrupted amygdala-prefrontal connectivity during emotion regulation links stress-reactive rumination and adolescent depressive symptoms. Developmental Cognitive Neuroscience, 27, 99–106. 28946039 10.1016/j.dcn.2017.09.002 PMC5626656

[ref32] Fukuda K., Vogel E.K. (2009). Human variation in overriding attentional capture. Journal of Neuroscience, 29(27), 8726–33. 19587279 10.1523/JNEUROSCI.2145-09.2009 PMC6664881

[ref33] Galvan A. (2010). Adolescent development of the reward system. Frontiers in Human Neuroscience, 4, 6. 20179786 10.3389/neuro.09.006.2010 PMC2826184

[ref34] Green D.M., Swets J.A. (1966). Statistical decision theory and psychophysical procedures. Signal Detection Theory and Psychophysics, Original ed, 40–3.

[ref35] Guassi Moreira J.F., Telzer E.H. (2018). Mother still knows best: maternal influence uniquely modulates adolescent reward sensitivity during risk taking. Developmental Science, 21(1), e12484. 10.1111/desc.12484 PMC5752112 29282834

[ref36] Guyer A.E., Monk C.S., McClure-Tone E.B., et al. (2008). A developmental examination of amygdala response to facial expressions. Journal of Cognitive Neuroscience, 20(9), 1565–82. 18345988 10.1162/jocn.2008.20114 PMC2902865

[ref37] Guyer A.E., Choate V.R., Pine D.S., Nelson E.E. (2011). Neural circuitry underlying affective response to peer feedback in adolescence. Social Cognitive and Affective Neuroscience, 7(1), 81–92. 21828112 10.1093/scan/nsr043 PMC3252630

[ref38] Hampshire A., Chamberlain S.R., Monti M.M., Duncan J., Owen A.M. (2010). The role of the right inferior frontal gyrus: inhibition and attentional control. NeuroImage, 50(3), 1313–9. 20056157 10.1016/j.neuroimage.2009.12.109 PMC2845804

[ref39] Hoorn J., Shablack H., Lindquist K., Telzer E. (2019). Incorporating the social context into neurocognitive models of adolescent decision-making: a neuroimaging meta-analysis. Neuroscience and Biobehavioral Reviews, 101, 129–42. 31006540 10.1016/j.neubiorev.2018.12.024 PMC6659412

[ref40] Humphreys K.L., Lee S.S., Tottenham N. (2013). Not all risk taking behavior is bad: associative sensitivity predicts learning during risk taking among high sensation seekers. Personality and Individual Differences, 54(6), 709–15. 23935235 10.1016/j.paid.2012.11.031 PMC3735177

[ref41] Hyde L.W., Shaw D.S., Hariri A.R. (2013). Understanding youth antisocial behavior using neuroscience through a developmental psychopathology lens: review, integration, and directions for research. Developmental Review, 33(3), 168–223. 10.1016/j.dr.2013.06.001 PMC3834895 24273368

[ref42] IBM Corp (2017). IBM SPSS Statistics for Windows, Version 25.0, Armonk, NY: IBM Corp.

[ref43] Juvonen J., Graham S., Schuster M.A. (2003). Bullying among young adolescents: the strong, the weak, and the troubled. Pediatrics, 112(6), 1231–7. 14654590 10.1542/peds.112.6.1231

[ref44] Kranzler A., Young J.F., Hankin B.L., Abela J.R., Elias M.J., Selby E.A. (2016). Emotional awareness: a transdiagnostic risk factor for internalizing symptoms in children and adolescents? Journal of Clinical Child and Adolescent Psychology, 45(3), 262–9. 25658297 10.1080/15374416.2014.987379 PMC4527953

[ref45] Lee N.C., Weeda W.D., Insel C., Somerville L.H., Krabbendam L., Huizinga M. (2018). Neural substrates of the influence of emotional cues on cognitive control in risk taking adolescents. Developmental Cognitive Neuroscience, 31, 20–34. 29729493 10.1016/j.dcn.2018.04.007 PMC6969196

[ref46] Lee T.H., Perino M.T., McElwain N.L., Telzer E.H. (2019). Perceiving facial affective ambiguity: a behavioral and neural comparison of adolescents and adults. Emotion. Advance online publication. 10.1037/emo0000558 PMC6620165 30628818

[ref47] McCormick E.M., Telzer E.H. (2017). Adaptive adolescent flexibility: neurodevelopment of decision-making and learning in a risky context. Journal of Cognitive Neuroscience, 29(3), 413–23. 28129057 10.1162/jocn_a_01061 PMC5362273

[ref48] McCormick E., Telzer E.H. (2018). Not doomed to repeat: enhanced mPFC tracking of errors promotes adaptive behavior during adolescence. Journal of Cognitive Neuroscience, 30, 281–9. 29131744 10.1162/jocn_a_01206 PMC5797691

[ref49] McCormick E.M., Qu Y., Telzer E.H. (2016). Adolescent neurodevelopment of cognitive control and risk taking in negative family contexts. NeuroImage, 124, 989–96. 26434803 10.1016/j.neuroimage.2015.09.063 PMC4651739

[ref50] McCormick E.M., Perino M.T., Telzer E.H. (2018). Not just social sensitivity: adolescent neural suppression of social feedback during risk taking. Developmental Cognitive Neuroscience, 30, 134–41. 29518712 10.1016/j.dcn.2018.01.012 PMC6014584

[ref51] McLaren D.G., Ries M.L., Xu G., Johnson S.C. (2012). A generalized form of context-dependent psychophysiological interactions (gPPI): a comparison to standard approaches. NeuroImage, 61(4), 1277–86. 22484411 10.1016/j.neuroimage.2012.03.068 PMC3376181

[ref52] Meijers J., Harte J.M., Meynen G., Cuijpers P., Scherder E.J. (2018). Reduced self-control after three months of imprisonment; a pilot study. Frontiers in Psychology, 9, 69. 29449824 10.3389/fpsyg.2018.00069 PMC5799890

[ref53] Menon V., Uddin L.Q. (2010). Saliency, switching, attention and control: a network model of insula function. Brain Structure and Function, 214(5–6), 655–67. 20512370 10.1007/s00429-010-0262-0 PMC2899886

[ref54] Moffitt T.E., Caspi A. (2001). Childhood predictors differentiate life-course persistent and adolescence-limited antisocial pathways among males and females. Development and Psychopathology, 13(2), 355–75. 11393651 10.1017/s0954579401002097

[ref55] Monk C.S., McClure E.B., Nelson E.E., et al. (2003). Adolescent immaturity in attention-related brain engagement to emotional facial expressions. NeuroImage, 20(1), 420–8. 14527602 10.1016/s1053-8119(03)00355-0

[ref56] Nelson E.E., Leibenluft E., McClure E.B., Pine D.S. (2005). The social re-orientation of adolescence: a neuroscience perspective on the process and its relation to psychopathology. Psychological Medicine, 35(2), 163–74. 15841674 10.1017/s0033291704003915

[ref57] Nelson E.E., Jarcho J.M., Guyer A.E. (2016). Social re-orientation and brain development: an expanded and updated view. Developmental Cognitive Neuroscience, 17, 118–27. 26777136 10.1016/j.dcn.2015.12.008 PMC6990069

[ref58] Perino M.T., Miernicki M.E., Telzer E.H. (2016). Letting the good times roll: adolescence as a period of reduced inhibition to appetitive social cues. Social Cognitive and Affective Neuroscience, 11(11), 1762–71. 27445208 10.1093/scan/nsw096 PMC5091687

[ref59] Perino M.T., Moreira J.F.G., Telzer E.H. (2019). Links between adolescent bullying and neural activation to viewing social exclusion. Cognitive, Affective, & Behavioral Neuroscience, 1–12. 10.3758/s13415-019-00739-7 PMC6864266 31292887

[ref60] Pfeifer J.H., Masten C.L., Moore W.E., et al. (2011). Entering adolescence: resistance to peer influence, risky behavior, and neural changes in emotion reactivity. Neuron, 69(5), 1029–36. 21382560 10.1016/j.neuron.2011.02.019 PMC3840168

[ref61] Phillips R. (2012). The financial costs of bullying, violence and vandalism. Proceedings of the National Association of Secondary School Principals, 28–9.

[ref62] Rocque M., Paternoster R. (2011). Understanding the antecedents of the ‘school-to-jail’ link: the relationship between race and school discipline. Journal of Criminal Law and Criminology, 101(2), 633–66.

[ref63] Saxe R. (2006). Uniquely human social cognition. Current Opinion in Neurobiology, 16(2), 235–9. 16546372 10.1016/j.conb.2006.03.001

[ref64] Serences J.T., Shomstein S., Leber A.B., Golay X., Egeth H.E., Yantis S. (2005). Coordination of voluntary and stimulus-driven attentional control in human cortex. Psychological Science, 16(2), 114–22. 15686577 10.1111/j.0956-7976.2005.00791.x

[ref65] Sharp D.J., Bonnelle V., De Boissezon X., et al. (2010). Distinct frontal systems for response inhibition, attentional capture, and error processing. Proceedings of the National Academy of Sciences of the United States of America, 107(13), 6106–11. 20220100 10.1073/pnas.1000175107 PMC2851908

[ref66] Silva K., Shulman E.P., Chein J., Steinberg L. (2016). Peers increase late adolescents' exploratory behavior and sensitivity to positive and negative feedback. Journal of Research on Adolescence, 26(4), 696–705. 28453197 10.1111/jora.12219

[ref67] Somerville L.H., Hare T., Casey B.J. (2011). Frontostriatal maturation predicts cognitive control failure to appetitive cues in adolescents. Journal of Cognitive Neuroscience, 23(9), 2123–34. 20809855 10.1162/jocn.2010.21572 PMC3131482

[ref68] Stanislaw H., Todorov N. (1999). Calculation of signal detection theory measures. Behavior Research Methods, Instruments, & Computers, 31(1), 137–49. 10.3758/bf03207704 10495845

[ref69] Swann N.C., Cai W., Conner C.R., et al. (2012). Roles for the pre-supplementary motor area and the right inferior frontal gyrus in stopping action: electrophysiological responses and functional and structural connectivity. NeuroImage, 59(3), 2860–70. 21979383 10.1016/j.neuroimage.2011.09.049 PMC3322194

[ref70] Swick D., Ashley V., Turken U. (2008). Left inferior frontal gyrus is critical for response inhibition. BMC Neuroscience, 9(1), 102. 18939997 10.1186/1471-2202-9-102 PMC2588614

[ref71] Telzer E.H. (2018). “(under) Representation of cultural diversity and high-risk youth samples in research on adolescent motivational process” Symposium presented at the 6th Annual Flux Conference, Berlin, Germany.

[ref72] Theeuwes J. (2010). Top–down and bottom–up control of visual selection. Acta Psychologica, 135(2), 77–99. 20507828 10.1016/j.actpsy.2010.02.006

[ref73] Psychology Software Tools, Inc (2012). E-Prime, 2.0.

[ref74] Van Hoorn J., Van Dijk E., Güroğlu B., Crone E.A. (2016). Neural correlates of prosocial peer influence on public goods game donations during adolescence. Social Cognitive and Affective Neuroscience, 11(6), 923–33. 26865424 10.1093/scan/nsw013 PMC4884312

[ref75] Viding E., McCrory E.J. (2012). Genetic and neurocognitive contributions to the development of psychopathy. Development and Psychopathology, 24(3), 969–83. 22781866 10.1017/S095457941200048X

[ref76] Vossel S., Geng J.J., Fink G.R. (2014). Dorsal and ventral attention systems: distinct neural circuits but collaborative roles. The Neuroscientist, 20(2), 150–9. 23835449 10.1177/1073858413494269 PMC4107817

[ref77] Wald J., Losen D.J. (2003). Defining and redirecting a school-to-prison pipeline. New Directions in Youth Development, 99(99), 9–15. 10.1002/yd.51 14635431

[ref78] Ward B. D. (2000). Simultaneous inference for fMRI data.

[ref79] Watts A.L., Poore H.E., Waldman I.D. (2019). Riskier tests of the validity of the bifactor model of psychopathology. Clinical Psychological Science. 2167702619855035.

[ref80] Weiner B. (2003). The classroom as a courtroom. Social Psychology of Education, 6(1), 3–15.

[ref81] Welner M., O’Malley K.Y., Gonidakis J., Tellalian R.E. (2018). The depravity standard I: an introduction. Journal of Criminal Justice, 55, 1–11.

[ref82] Young S.E., Friedman N.P., Miyake A., et al. (2009). Behavioral disinhibition: liability for externalizing spectrum disorders and its genetic and environmental relation to response inhibition across adolescence. Journal of Abnormal Psychology, 118(1), 117–30. 19222319 10.1037/a0014657 PMC2775710

